# Subliminal Semantic Priming in Speech

**DOI:** 10.1371/journal.pone.0020273

**Published:** 2011-05-31

**Authors:** Jérôme Daltrozzo, Carine Signoret, Barbara Tillmann, Fabien Perrin

**Affiliations:** 1 CNRS, UMR5292, Lyon Neuroscience Research Center, Auditory Cognition and Psychoacoustics Team, Lyon, France; 2 INSERM, U1028, Lyon Neuroscience Research Center, Auditory Cognition and Psychoacoustics Team, Lyon, France; 3 University of Lyon, Lyon, France; University of Zaragoza, Spain

## Abstract

Numerous studies have reported subliminal repetition and semantic priming in the visual modality. We transferred this paradigm to the auditory modality. Prime awareness was manipulated by a reduction of sound intensity level. Uncategorized prime words (according to a post-test) were followed by semantically related, unrelated, or repeated target words (presented without intensity reduction) and participants performed a lexical decision task (LDT). Participants with slower reaction times in the LDT showed semantic priming (faster reaction times for semantically related compared to unrelated targets) and negative repetition priming (slower reaction times for repeated compared to semantically related targets). This is the first report of semantic priming in the auditory modality without conscious categorization of the prime.

## Introduction

The extent to which words can be processed unconsciously has been a topic of considerable debate. Unlike studies of implicit word processing [1], studies of subliminal word processing are rather rare. This might be due to the complex questions this research domain has to face: in particular, how to demonstrate the absence of consciousness and how to measure unconscious effects [2], [3]. In the visual modality, a method for studying subliminal processing consists in the presentation of stimuli in a subliminal priming paradigm: the prime word is presented for a short time (usually less than 50 ms) and is surrounded by a forward and/or a backward visual mask [4], [5], [6]. In these conditions, visual orthographic and morphological priming have been observed [5], [7]. Other studies have shown phonological and semantic effects with this paradigm [8]–[14], but some of them have received severe criticisms, notably because no index of prime awareness was provided (see [15], [16] for reviews).

In the auditory modality, subliminal perception has been considerably less investigated. Similarly to the visual modality, masking techniques (e.g., white noise) have been used to reduce prime awareness. However, most of these studies reported contradictory results [17], allowing no clear conclusions. More recently, by transferring the visual subliminal priming paradigm to the auditory domain, i.e. by using masked and time-compressed primes, Kouider and Dupoux [18] reported subliminal repetition priming for speech, but no subliminal semantic priming.

Studying auditory subliminal priming requires adaptations of the experimental design because of the sequential nature of speech presentation and because of longer processing times in the auditory compared to the visual domain. Unlike visual words, which can be fully presented in a short time window (without distortion), spoken words require time for presentation/pronunciation. In addition, processing is lengthened in the auditory domain as compared to the visual domain [19], [20], notably because of the greater number of relays in the ascending auditory pathway. This domain specificity suggests that in an auditory subliminal priming experiment, primes may not be fully perceived before the participants' response when (a) participants are asked to perform a task as fast as possible, (b) the duration between primes and targets is very short, and (c) primes are difficult to perceive. Hence, if a participant responds rather fast, priming might be attenuated because of an incomplete processing of the prime. This is in line with Wundt's [21] early prediction that auditory priming might be sensitive to participants' response speed and is consistent with more recent research [22]–[23]. For instance, phonological or conceptual priming studies have reported contextual facilitation for slow responders but not for fast responders when prime processing required a long processing time, either because of the auditory modality or because of the task difficulty [24]–[27].

Here, we investigated auditory subliminal repetition and semantic priming by using primes presented at low intensity, i.e. in competition with the internal noise (i.e. the random variability in participants' neural responses to sensory stimuli [28], [29]). We took into account the specificity of the auditory domain by contrasting fast- with slow-responders' performance and we predicted faster response-times (in a lexical decision task) to semantically primed target words presented at low sound intensity for the group of slow-responders.

After the priming experiment, a prime categorization test (word/pseudo-word) was presented to check that participants were unable to categorize the low-intensity primes. The prime categorization was used as a measure of prime awareness, as in previous studies [11]–[14], [30], including those investigating the auditory modality [18].

## Materials and Methods

### Participants

Forty-five volunteers were tested: 16 in a pre-experiment (12 females, 22.1±0.4 years) and 29 others in the main experiment. All participants were right-handed according to the Edinburgh Handedness Inventory [31], native French speakers, and did not report any hearing problems or history of neurological disease. All participants provided written informed consent to the study, which was conducted in accordance with the guidelines of the Declaration of Helsinki, and approved by the local Ethics Committee (CPP Sud-Est II). Participants of the main experiment had pure tone auditory thresholds below 15 dB-HL for frequencies between 250 Hz to 8000 Hz [32]. In the main experiment, a lexical decision task (LDT) on primed targets was followed by two post-tests. Five participants were excluded from the main experiment because of their poor LDT performance or because their performance differed by more than two standard deviations (SD) from the group performance of the post-tests (see details at the end of the Procedure section). Thus, 24 participants (16 females, 21.5±0.3 years) were included in the analysis of the main experiment.

### Stimuli

One hundred and sixty words were selected from a French database (Lexique 2, [33]). They were monosyllabic nouns of two to seven letters and two to five phonemes (e.g., “sable” [sand], “vache” [cow]). All words had a frequency of occurrence higher than one per million occurrences in books and in movies (subtitles). A list of monosyllabic pseudo-words was created using all phonemes of the words, the number of phonemes being matched to the words. Pseudo-words could be pronounced, but were meaningless (according to a pre-experiment). The average durations of words and pseudo-words were 521 ms (SD = 115 ms) and 539 ms (SD = 88 ms), respectively. To reduce differences in the perceived loudness, all stimuli were equalized to reach the same dB-A level (A-weighting roughly mimics the external and middle ear transfer functions; [34]).

Words and pseudo-words were uttered by the same female speaker and recorded at 32 bits and 44.1 kHz. The mean level of presentation was calibrated with a standard artificial ear to reach 80 dB-A. A null (for primes and targets of the pre-experiment and for targets of the main experiment) or moderate digital attenuation (35 dB for primes of the main experiment) was combined with an analog fixed attenuation. This attenuation was analog rather than digital to prevent acoustic distortion at low levels of presentation. All stimuli were binaurally presented to participants through headphones.

### Procedure

#### Pre-experiment

To check that repetition priming and semantic priming were elicited with our experimental material, a first group of participants heard a (prime) word, followed by a (target) word or pseudo-word. Participants were asked to decide whether the target was a word or a pseudo-word (i.e. performed a LDT). Primes and targets were presented at a comfortable hearing level (60 dB-A) to sixteen participants. They performed a LDT on the target as fast and accurately as possible by pressing one of two buttons. One hundred and twenty prime-target pairs were presented in random order: sixty with a word target (20 semantically related to the prime (categorically or associatively related), 20 semantically unrelated to the prime, and 20 repeated) and sixty with a pseudo-word target. Across participants, words were used as either semantically related, semantically unrelated, or repeated. For each participant, none of the words was repeated, except inside the pairs of the repeated condition. A fixation cross was displayed in the center of a monitor screen while the prime word was presented. The target was presented 50 ms after the end of the prime (and of the fixation cross).

#### Main experiment

A second group of participants performed first the LDT in a subliminal priming paradigm and then two post-tests: a prime detection task and a prime categorization task.

The priming phase was the same as in the pre-experiment, except for the sound level (prime: 10 dB-A; target: 45 dB-A) and the instructions: participants were not told about the presence of the prime. The 10 dB-A intensity level was chosen because previous experiments by our team suggested an absence of conscious categorization at this level (Signoret, Tillmann, Gaudrain, Grimault, & Perrin. Facilitated auditory detection for speech. *Submitted*).

Awareness of the prime was estimated with a prime categorization post-test, as a standard measure of prime awareness (e.g., [18] in the auditory modality, and [13], [30], [35]–[37] in the visual modality). A word (n = 60) or a pseudo-word (n = 60), was randomly presented at 10 dB-A together with the fixation cross. Participants were told to decide whether a word or a pseudo-word was presented during the fixation cross and to give their response as accurately as possible and as soon as the fixation cross had disappeared. Participants were told that the task was difficult and that they should not be discouraged by the difficulty. Fifty milliseconds after the participant's response, a second stimulus (a word or a pseudo-word), for which no task was requested, was presented at 45 dB-A to maintain the same intensity context as in the priming phase [38]. None of the stimuli were repeated between the two phases of the main experiment, but across participants all stimuli presented in the priming phase were used in the post-test.

Since our aim was to study subliminal auditory perception in a homogenous participant group, participants had to be (1) able to perform the LDT in the priming phase of the main experiment, (2) unable to categorize the prime, but nevertheless (3) able to detect it. To test for prime detection, participants performed a detection task (present/absent) in which a word (n = 60) or a silence (n = 60) was randomly presented at the same time as a fixation cross. To avoid participants searching for words, this prime detection task was performed before the prime categorization task. Analysis of the prime detection task showed an average accuracy of 87.4±1.7% and a *d′* sensitivity (computed according to the Signal Detection Theory (SDT), [39]) of detection (*d′*D) of 2.7±0.1. Five participants were excluded from the analysis of the main experiment because (1) their performance at the LDT in the priming phase of the main experiment were below the group's mean accuracy minus two SD, or (2) their performance at the prime categorization task were two SD above the group mean *d′* sensitivity of categorization (*d′*C) (close to zero, i.e., chance level), or (3) their detection of the prime was two SD below the group mean *d′*D. Priming is assumed to be subliminal when the performance on the target is above zero, but the performance on the prime is null (here at *d′*C = 0) [41]. When this is not the case (or for confirmation purposes), Greenwald, Klinger, and Schuh [36] introduced a regression method that allows investigating whether the priming is still reliable when the performance on the prime is extrapolated to zero. Subliminal priming would be shown when, the estimated priming at *d′*C = 0 (corresponding to the y-intercept of the regression) is significantly different from zero. *d′*C was computed for each participant. Thus, the regression was based on a sample size equal to the participant sample size.

## Results

Accuracy and correct RTs were analyzed with ANOVAs using Relatedness (3 levels: related, unrelated, and repeated word pairs) as within-subject factor and Rapidity (slow responders/fast responders) as between-subjects factor. Participants were separated into slow and fast responders with a median split. All reported *p*-values were adjusted with Greenhouse–Geisser correction for nonsphericity when appropriate. Fisher's Least Significant Difference test was applied for post-hoc comparisons as the Relatedness factor had only three levels [40].

### Pre-experiment

**Figure 1:**
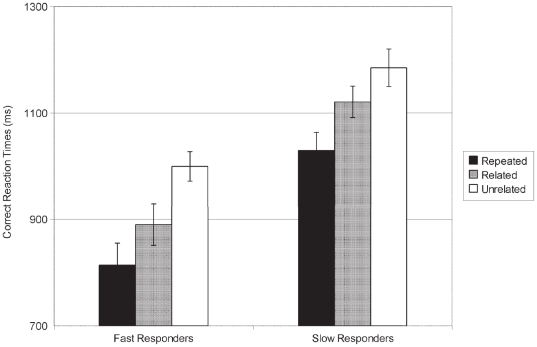
Correct Reaction Times at the lexical decision task – pre-experiment. Correct Reaction Times at the lexical decision task in the pre-experiment with semantically unrelated, semantically related, and repeated word pairs in slow (N = 12) and fast responders (N = 12) (unit: milliseconds; with SEM).

Average LDT accuracy was 96.2±0.7%. There was a main effect of Relatedness [*F*(2,28) = 6.90; *p* = .004; η*p* ^2^ = .330] with better accuracy for semantically related (98.7±0.6%) and repeated word pairs (97.5±0.6%) than for unrelated pairs (92.5±2.2%) (*ps*<.01). There was no main effect of Rapidity (slow/fast) [*p* = .22] and no interaction with Relatedness [*p* = .12]. For RTs (**Figure 1**), the effect of Relatedness was also significant [*F*(2,28) = 21.8; *p*<.001; η*p* ^2^ = .609]: RTs were faster for repeated targets (921±38 ms) than for semantically related (1005±38 ms) (*p* = .003) and unrelated targets (1092±32 ms) (*p*<.001); RTs were also faster for semantically related versus unrelated targets (*p* = .002). A main effect of Rapidity [*F*(1,14) = 28.8; *p*<.001; η*p* ^2^ = .673] confirmed that slow and fast responders' RTs were significantly different. No interaction between Rapidity and Relatedness was observed [*p* = .67]. These results indicated that our material, when both primes and targets were presented at a comfortable hearing level (60 dB-A), elicited the expected semantic and repetition priming effects.

### Main experiment

**Figure 2:**
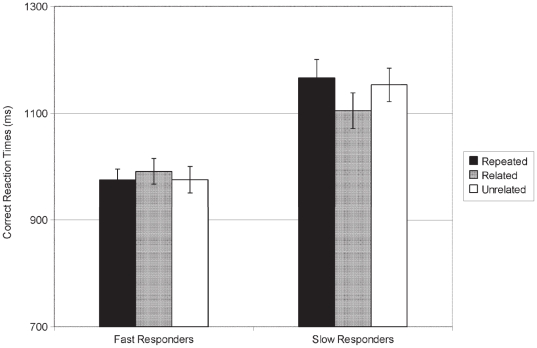
Correct Reaction Times at the lexical decision task - priming phase of the main experiment. Correct Reaction Times at the lexical decision task in the priming phase of the main experiment with semantically unrelated, semantically related, and repeated word pairs in slow (N = 12) and fast responders (N = 12) (unit: milliseconds; with SEM).

In the priming phase, the overall LDT accuracy was high (93.9±0.7%) and there was no significant effect of Relatedness nor an interaction between Relatedness and Rapidity (*ps*>.17). For correct RTs (**Figure 2**), the interaction between Relatedness and Rapidity (slow/fast) was significant [*F*(2,44) = 3.54; *p* = .04; η*p* ^2^ = .139]. For slow responders only, RTs were smaller for semantically related targets (1105±29 ms) than for unrelated (1153±28 ms) (*p* = .034) and repeated targets (1166±28 ms) (*p* = .008). RTs did not differ between unrelated and repeated targets (*p* = .549). For the fast responders, no significant differences were observed (*ps*>.470). In addition, a main effect of Rapidity [*F*(1,22) = 19.8; *p*<.001; η*p* ^2^ = .474] confirmed that slow and fast responders' RTs were significantly different.

In the post-test prime categorization task, the average accuracy was 52.0±1.2% (chance level: 50%) with *d′*C = .10±.04 (slow group: 51.6±1.6% with *d′*C = .08±.05; fast group: 52.4±1.8% with *d′*C = .12±.06). The *d′*C was not significantly greater than zero for the slow group [*t*(11) = 1.58, *p* = .14] and the fast group [*t*(11) = 1.94, *p* = .08] and did not differ significantly between the fast and slow groups [*t*(22) = .51, *p* = .61], thereby suggesting that participants were unable to categorize the prime.

**Figure 3:**
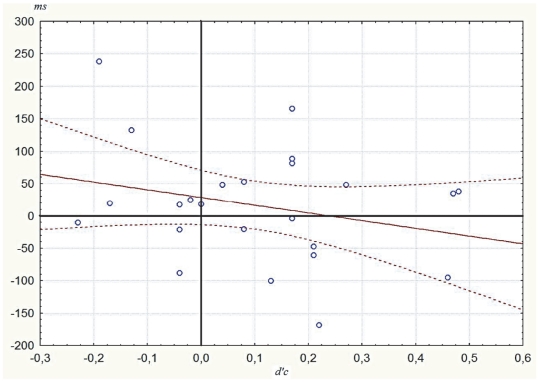
Regression line of the priming between related and unrelated pairs. Regression line of the priming between related and unrelated pairs (vertical axis: correct reaction time difference between the two conditions in milliseconds) as a function of the prime awareness sensitivity *d′c* (see Methods), on the horizontal axis.

**Figure 4:**
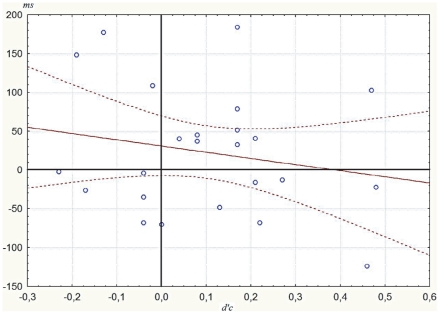
Regression line of the priming between related and repeated pairs. Regression line of the priming between related and repeated pairs (correct reaction time difference between the two conditions in milliseconds) as function of the prime awareness sensitivity *d′c* (see Methods), on the horizontal axis.

As categorization sensitivity for the entire group was slightly greater than zero (*d′C* = .10±04, *t*(22) = 2.54, *p* = .02), priming at *d′C* = 0 was estimated through regression analyzes [41]. The regression line of priming effects between related and unrelated pairs was *y* = −120x+29 with a *y*-intercept that was significantly above zero [*t*(22) = 2.77, *p* = .010] (**Figure 3**). According to this regression, at *d′C* = 0, participants would respond 29 ms faster to related pairs compared to unrelated pairs and this RT difference would be significant (*p* = .010). The regression line of priming effects between related and repeated pairs was *y* = −80.2x+31.0 with a *y*-intercept that was significantly above zero [*t*(22) = 2.70, *p* = .012] (**Figure 4**). According to this regression, at *d′C* = 0, participants would respond 31 ms faster to related pairs compared to repeated pairs and this RT difference would be significant (*p* = .012). As these estimated priming effects at *d′C* = 0 (corresponding to the y-intercepts) differed from zero, these regression suggested semantic and negative repetition priming when participants were not able to categorize the stimuli.

## Discussion

By reducing the sound intensity of prime words, our study suggests semantic speech priming in the absence of awareness (as measured by a prime categorization task). Participants were faster to discriminate target words that followed semantically related words than target words that followed semantically unrelated words. Moreover, they showed faster responses for semantically related words than for repeated words (a negative repetition priming). These two priming effects were particularly well observed for slow responders.

### Subliminal semantic priming in speech

Our study is the first study to report subliminal semantic priming with auditory primes. In the visual domain, only a few studies that properly controlled the level of prime awareness have reported unconscious semantic priming effects [16]. Dell'Acqua and Grainger [12] showed a 30-ms faster target word categorization when the unconsciously perceived picture prime belonged to the same semantic category (compared to a different semantic category). They further replicated their findings with target pictures and a picture-naming task, showing a 22-ms unconscious facilitation in the semantically related condition. Using visual word pairs and a LDT, Kiefer and Brendel [14] reported unconscious semantic priming with RT differences of about 30 ms (see Figure 5 in [14]) and with Event-Related Potentials. Furthermore, with visual prime numbers and auditory or visual target numbers, Kouider and Dehaene reported a subliminal number priming [4]. They interpreted their results as based on semantic or sensorimotor priming (for a discussion about semantic and sensorimotor priming interpretations, see [15]).

Interestingly, the size of the auditory semantic priming effect we report here (48 ms) is larger than what has been observed in these studies. This difference might be due to the modality. At least under conscious perception, larger semantic priming can be found in the auditory modality than in the visual modality [19]. This difference might be further explained by a difference in the procedure for prime awareness reduction. In two of these prior studies [12], [14], the visual prime was presented briefly between a forward and a backward mask. Although the visual prime was not physically degraded, the presentation of these two masks in close temporal conjunction might have resulted in a single percept of a degraded prime [41]. Possibly, a physical or perceptual degradation of the prime might reduce the activation of its representation in the mental lexicon and yield weaker priming. In contrast, the primes of our present study were neither degraded physically nor at a perceptual level (the only degradation might have resulted from the internal noise of the perceiver).

In the auditory modality, subliminal semantic priming has never been reported up to now. The absence of semantic priming in Kouider and Dupoux [18] might be explained by the fact that they used time-compressed and masked primes and/or that they did not analyze their results separately for slow and fast responders. It is possible that unconscious semantic priming was present in their study, but was reduced by the time distortion of the prime. Indeed, Beattie [42] has shown a decrease of intelligibility with 60% time-compressed speech, suggesting impaired semantic analyzes. Furthermore, as everyday life speech perception usually does not require the perception of time-compressed words, it is likely that stimuli of this type have a poor representation in the mental lexicon or lead to a poor access to this lexicon, resulting in weak semantic priming.

### Subliminal repetition priming in speech

Participants responded faster to target words that followed semantically related prime words than to target words that repeated the prime, an effect referred to as negative priming [43]. This priming was comparable to an effect reported for compatible trials [44], notably when the awareness of the prime was reduced [45]. In our study, the negative priming effect was observed for repeated words when the awareness of the prime was reduced, whereas under awareness (in our pre-experiment), the same experimental material showed the classical repetition effect (with strongest facilitation for repeated targets, see [46] for a review).

One explanation of negative repetition priming is that inhibitory mechanisms might affect RTs. Eimer and Schlaghecken [44] proposed inhibitory mechanisms to account for this “negative compatibility effect” (or “inverse priming”) that had been observed for repeated pairs of visual arrows. The negative compatibility effect has been replicated several times and would occur at the perceptual processing stage, i.e., before response decision [47]. The reason for the emergence of this inhibitory mechanism is still being debated [48], [49]. One possible explanation is based on the influence of a backward mechanism [50]. According to Kahan's Retrospective Prime Clarification theory, an inhibitory mechanism could result from a memory retrieval process that compares the activated (semantic, orthographic, and phonological) representations of the target with the memory trace of the prime [50]. Both “forward” semantic priming (i.e. when the semantic processing of the prime influences the perception of the target) and “backward” semantic priming (i.e. when the semantic perception of the target influences the processing of the prime) must be taken into account in the present study. While “forward” semantic priming reflects facilitatory mechanisms, as shown by positive priming, “backward” semantic priming often leads to a negative priming that reflects inhibitory mechanisms [51]. Repetition priming would arise from the combined effects of positive forward priming (or facilitation) and negative backward priming (or inhibition). In our study, this negative backward priming was likely to occur during both the pre-experiment (i.e., when the prime and the target were presented supraliminally) and during the main experiment (i.e., when primes were presented subliminally and targets supraliminally) because backward priming requires the perception of the target, which was presented supraliminally in these two experiments. What was likely to change the most between the pre-experiment and the main experiment is the strength of the positive forward priming, which requires the perception of the prime. If the positive (forward) priming is stronger than the negative (backward) priming, as suggested by the overall positive priming in the pre-experiment when neither the forward nor the backward priming were attenuated (thanks to the supraliminal presentation), the overall negative priming observed in the main experiment suggests that the positive (forward) priming was attenuated (the prime perception being subliminal) while the negative (backward) priming remained strong (the target perception being supraliminal).

The lack of RTs difference between unrelated and repeated targets (see Figure 2) would thus result from the positive (forward) priming (between the repeated words) being canceled out by the negative (backward) priming.

In contrast to the negative repetition priming observed here, Kouider and Dupoux [18] have shown positive repetition priming. Possibly, the above-mentioned inhibitory mechanisms, which explain negative repetition priming, were attenuated in their study because of stronger physical (acoustic) differences between the target and the prime as compared to our study. Indeed, these authors used a different procedure to reduce prime awareness. While they time-compressed the prime and surrounded the prime with masks, we did not degrade the prime, but instead presented the prime at a low intensity level (where only degradation due to participants' internal noise may have occurred). Provided that the negative (backward) repetition priming is highly sensitive to physical differences between the target and the prime, this priming might have been more attenuated in their study compared to ours. In addition, since the prime awareness was larger in Kouider and Dupoux's study (with a d′' of 0.21 and of 0.24 in their two subliminal conditions) compared to our study (with a d′' = 0.10 in our subliminal condition, thus two-times smaller) the positive (forward) priming might have been less attenuated in their study than in ours. Since, the negative repetition priming could be smaller and the positive repetition priming stronger in Kouider and Dupoux's study compared to our study, the addition of the negative and positive repetition priming (i.e., the overall repetition priming) could be positive in their study and negative in ours.

### Rapidity effect

Even though regression analyzes suggest subliminal priming in all our participants, the RT data showed these effects only in the group of slow responders. This could be a confirmation of our prediction that a differential facilitation between fast- and slow-reacting participants would occur because the processing time of the subliminally presented primes would be lengthy and difficult. Slow responders took more time than fast responders to achieve matching processes between the prime and the target [31]. Within the model of parallel contingent processing of Milner [52], fast-responders would only transmit partial stimulus-evaluation to the response-preparation process [53]–[58] while slow-responders would wait for a more complete stimulus evaluation before response preparation.

In contrast, when participants were aware of the prime, priming did not differ between slow and fast responders. These results suggest that participants need more time and cognitive load to process the prime when it is presented subliminally as compared to when it is presented supraliminally.

### Measures of awareness

As done by Kouider and Dupoux [18] in the auditory domain and other researchers in the visual domain [11]–[14], [30], we controlled the awareness of the prime with a categorization task. In our study, and probably in previous studies using categorization as the measure of awareness, priming effects are observed while primes are still detected (i.e., visible or audible). The definition of the best measure of awareness is still a matter of debate. According to Merikle and Reingold [59], a valid measure should meet the criteria of being exhaustive (i.e., sensitive to conscious experience so that no residual conscious perception goes unmeasured) and exclusive (i.e., representing only conscious perception rather than a combination of conscious and unconscious perception). While it seems difficult to meet the exclusiveness criterion (see [3]), the criterion of exhaustivity appears to be within reach. A measure of awareness based on detection tasks (i.e., asking for the presence or absence of the stimulus) is known for its greater sensitivity compared to a measure of awareness based on discrimination tasks. According to Snodgrass and colleagues [60], the detection task would be the best candidate (as compared to identification or semantic classification tasks) for meeting the exhaustiveness criterion. In contrast, a measure of awareness based on discrimination tasks may lack exhaustivity because of partial awareness of non-discriminated stimuli (that nevertheless might be detected). Our choice of a semantic classification tasks (lexical decision) following Kouider and Dupoux [18] appears however to be the best within a semantic priming paradigm. Obviously, when trying to show unconscious semantic priming, one needs to control the awareness of the semantic meaning of the stimuli, not the purely physical awareness of the stimulus presence, that may be reached without awareness of the stimulus semantic meaning.
